# Cytoskeleton-Associated Protein 4, a Promising Biomarker for Tumor Diagnosis and Therapy

**DOI:** 10.3389/fmolb.2020.552056

**Published:** 2021-02-05

**Authors:** Shuang-Xi Li, Juan Li, Li-Wei Dong, Zhi-Yong Guo

**Affiliations:** ^1^ Department of Nephrology, Changhai Hospital, The Navy Military Medical University, Shanghai, China; ^2^ International Cooperation Laboratory on Signal Transduction, Eastern Hepatobiliary Surgery Hospital, The Navy Military Medical University, Shanghai, China; ^3^ National Center for Liver Cancer, Shanghai, China

**Keywords:** CKAP4, receptor, tumorigenesis, serological marker, tumor therapy

## Abstract

Cytoskeleton-associated protein 4 (CKAP4) is located in the rough endoplasmic reticulum (ER) and plays an important role in stabilizing the structure of ER. Meanwhile, CKAP4 is also found to act as an activated receptor at the cell surface. The multifunction of CKAP4 was gradually discovered with growing research evidence. In addition to the involvement in various physiological events including cell proliferation, cell migration, and stabilizing the structure of ER, CKAP4 has been implicated in tumorigenesis. However, the role of CKAP4 is still controversial in tumor biology, which may be related to different signal transduction pathways mediated by binding to different ligands in various microenvironments. Interestingly, CKAP4 has been recently recognized as a serological marker of several tumors and CKAP4 is expected to be a tumor therapeutic target. Therefore, deciphering the gene status, expression regulation, functions of CKAP4 in different diseases may shed new light on CKAP4-based cancer diagnosis and therapeutic strategy. This review discusses the publications that describe CKAP4 in various diseases, especially on tumor promotion and suppression, and provides a detailed discussion on the discrepancy.

## Introduction

CKAP4 is an intriguing protein. From the initial studies of its molecular structure to the gradual recognition of its biological properties and functions, more and more researches have shown that CKAP4 has a relationship with various types of tumor. It is expected to be a biological marker for the diagnosis and molecular target of tumors.

Cytoskeleton-associated protein 4 (CKAP4), discovered in the early 1990s (Schweizer et al., 1993a), also known as cytoskeleton-linking membrane protein (CLIMP-63) and p63, is a reversibly palmitoylated but nonglycosylated type II transmembrane protein (Schweizer et al., 1993a; Schweizer et al., 1993b; Schweizer et al., 1994). CKAP4 was originally identified as a resident protein in the membrane network between Golgi apparatus and rough endoplasmic reticulum (ER) (Schweizer et al., 1993a; Schweizer et al., 1993b). Later researches confirmed that CKAP4 was located in the rough ER (Schweizer et al., 1995b).

It was observed that the luminal domain of CKAP4 has the capacity to form clusters in the ER (Schweizer et al., 1993a; Schweizer et al., 1994) and cause multimerization which constitutes the ER sheets and affects the mobility of ER membrane (Klopfenstein et al., 2001; Barlowe, 2010). Thus, CKAP4 plays an important role in stabilizing the structure of ER. On the other hand, CKAP4 is thought to directly mediate the interaction of ER with microtubules. CKAP4 was reported to bind microtubules which induced a rearrangement of the ER and microtubule networks, and this process was negatively regulated by phosphorylation (Schweizer et al., 1995b; Klopfenstein et al., 1998; Vedrenne et al., 2005). CKAP4-mediated microtubule-ER binding affects the lateral mobility of the translocon complexes and that may play an important role in segregating the rough and smooth domains of the ER (Nikonov et al., 2007). Another report supported the association of CKAP4 with ER-bound ribosomes, showing that CKAP4 relocalized from the rough ER to the entire ER (except for the nuclear membrane) under treatment of cells with puromycin which disassembles the polysomes (Shibata et al., 2010).

CKAP4 presents not only in ER, but also at the plasma membrane (Razzaq et al., 2003; Conrads et al., 2006; Gupta et al., 2006; Barbier et al., 2012; Kimura et al., 2016; Kajiwara et al., 2018). Intriguingly, CKAP4 has been identified as a receptor for tissue plasminogen activator (tPA) (Razzaq et al., 2003), surfactant protein A (SP-A) (Gupta et al., 2006), antiproliferative factor (APF) (Conrads et al., 2006), alginate exopolysaccharides (Barbier et al., 2012), and Dickkopfs (DKKs) protein (Kimura et al., 2016; Kajiwara et al., 2018). Binding to different ligands determines the different roles elicited by CKAP4 protein. It has been reported that CKAP4 is a protumor molecule to promote progression of various cancers, such as pancreatic, lung, esophageal, and renal tumors (Kimura et al., 2016; Sun et al., 2017; Kajiwara et al., 2018; Shinno et al., 2018). However, there are opposing reports about CKAP4 as an anticancer protein in various kinds of tumors, such as hepatocellular carcinoma (HCC), intrahepatic cholangiocellular carcinoma (ICC), and glioma (Li et al., 2013; Li et al., 2014b; Lu et al., 2018). Whether CKAP4 is an anticancer or a procancer molecule still needs further exploration. Recent studies have found that CKAP4 can be detected in the serum of HCC, pancreatic ductal adenocarcinoma, esophageal squamous cell carcinoma, and lung cancer patients (Li et al., 2016; Cheng et al., 2017; Yanagita et al., 2018; Kimura et al., 2019; Wang et al., 2019). Therefore, CKAP4 is expected to become a novel serological marker for tumor diagnosis.

Except for cancer diseases, CKAP4 has been related to noncancers, such as drug-induced cytotoxicity (Karasawa et al., 2010) and interstitial cystitis/painful bladder syndrome (IC/PBS) (Conrads et al., 2006). After more than 20 years of research, the structures and functions of CKAP4 are gradually recognized. The purpose of this review is to summarize published evidence to understand this mysterious protein in multiple dimensions.

## Structure of CKAP4

**FIGURE 1:**
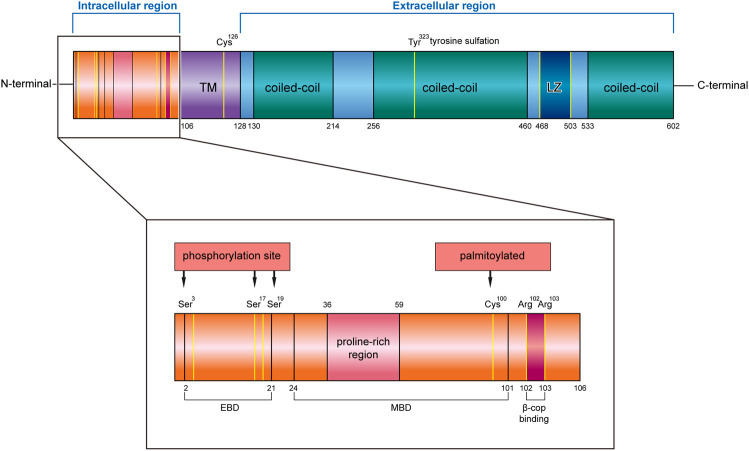
Structure characteristics of human CKAP4 protein. It contains three regions, an intracellular region, TM region, and an extracellular region. The intracellular region is enlarged, showing important amino acids site or domain. EBD, ER binding domain; MBD, microtubule binding domain; TM, transmembrane; LZ, leucine zipper; Ser, serine; Cys, cysteine; Tyr, tyrosine; Arg, arginine.

CKAP4 has three regions in humans, an intracellular domain of 105 amino acids (AA 1–105), a single transmembrane region of 22 amino acids (AA 106–127), and an extracellular region of 475 amino acids (AA 128–602) (Klopfenstein et al., 2001) (Figure 1).

The intracellular region contains a proline-rich region (AA 36–59) followed by seven glycine, six serine, and eight alanine repeats. Mutational analysis demonstrated that the cytoplasmic domain of CKAP4 carries two functionally distinct determinants: ER-anchoring domain (AA 2–21) and a microtubule binding domain (AA 24–101) (Schweizer et al., 1994) (Figure 1). The minimal identified sequence necessary for binding microtubules, while conserving ER localization, was residues 62–101 (Vedrenne et al., 2005). In terms of the described effects of CKAP4 on the architecture of the ER and microtubule networks, there are predicted phosphorylation sites of serine 3, 17, and 19 on CKAP4 (Figure 1), which have shown negatively regulated microtubule and induced collapse of the ER around the nucleus (Vedrenne et al., 2005; Vedrenne and Hauri, 2006). The function of proline-rich region on CKAP4 is associated with AKT activation, mediated by forming a complex with SH3 domain of p85α under DKK1 inducement (Kimura et al., 2016). Recent research showed that the intracellular domain (AA 1–21) of CKAP4 was crucial for binding β1 integrin (Osugi et al., 2019). In this region, the amino acids 102 and 103 were also found to be possible binding sites for β-COP (Bates, 2010) (Figure 1).

In the transmembrane region, it contains an important site; the cysteine in position 126 (Cys126) is a target of the disulfide bond, which makes CKAP4 becoming a dimer (Kikuchi et al., 2017; Schweizer et al., 1995a). Near the region, it has another important cysteine residue in position 100 (Cys100) (Figure 1), which is vital for palmitoylation of CKAP4. Palmitoyl acyltransferase DHHC2, which is a putative tumor suppressor, modifies Cys100 of CKAP4 (Schweizer et al., 1993b; Zhang et al., 2008) and contributes to the localization of CKAP4 on the plasma membrane (Planey et al., 2009).

In the extracellular region, CKAP4 contains three coiled-coil regions, two shorter regions (AA 130–214 and AA 533–602) and one longer one (AA 256–460), and a leucine zipper (LZ) domain (AA 468–503) (Figure 1). The coiled-coil regions of CKAP4 play an important role in the assembly of the ordered rough ER in the active secretory cells (Barlowe, 2010). In muscle cells, the luminal coiled-coil domain of CKAP4 interacts with triadin and reorganizes the microtubule network (Osseni et al., 2016). Furthermore, these coiled-coil domains are also required for its multimerization and intermolecular interactions (Klopfenstein et al., 2001; Schweizer et al., 1993a), which associate with tyrosine sulfation of CKAP4. Tyrosine 323 in one-third of the large coiled region is part of a predicted consensus tyrosine sulfation sequence (Figure 1). The position between the helical regions is ideal for interactions with other proteins. The leucine zipper region is also located in coiled regions, between the second and the third coil. The luminal domain (AA 192–258) of CKAP4 was recently reported to interact with the ribonuclease dicer (Pepin et al., 2012). Dicer is glycosylated and can be secreted into the extracellular environment by binding with CKAP4. The CKAP4–dicer complex showed a pre-microRNA processing activity (Pepin et al., 2012). The extracellular domain (AA 127–524) of CKAP4 is required for antiproliferative factor (APF) binding, which suggests that targeting this special region of CKAP4 to inhibit APF binding may be an effective strategy for the treatment of interstitial cystitis related to bladder pathology (Chavda et al., 2017).

## CKAP4 as a Surface Receptor

Although CKAP4 is mainly located in the ER, several studies report the presence of CKAP4 at the plasma membrane during the last decade. CKAP4 has been shown to be as a receptor of tissue plasminogen activator (tPA) (Razzaq et al., 2003), surfactant protein A (SP-A) (Gupta et al., 2006), APF (Conrads et al., 2006), and DKK family proteins (Kimura et al., 2016; Kajiwara et al., 2018), respectively (Table 1).

**TABLE 1:** CKAP4 functions as a cell surface membrane protein.

Cell type	Ligand	Functions
VSMCs	tPA	Progression of fibrinolysis
Type II pneumocytes	SP-A	Surfactant turnover
AEC	Alginate exopolysaccharides	Inflammation
Bladder epithelial cells	APF	Inhibits cell proliferation
MDCK epithelial cells	DKKs	Cell proliferation, others?

AEC: airway epithelial cells; MDCK, Madin-Darby canine kidney; SP-A: surfactant protein A; tPA: tissue plasminogen activator; VSMCs: vascular smooth muscle cells.

### Tissue Plasminogen Activator

CKAP4 was confirmed to present at the surface of vascular smooth muscle cells (VSMC) through using specific monoclonal antibody to probe human VSMC by immunofluorescence microscopy (Razzaq et al., 2003). CKAP4 binds to tPA on the surface of VSMC and anti-CKAP4 antibody abolished this interaction between tPA and VSMC (Razzaq et al., 2003), which suggested that CKAP4 was the functional tPA binding site on VSMC. A truncation mutant of CKAP4, which lacks the domain of 2–101AA (the sequence necessary for retention in the ER), has been shown to only transport to plasma membrane (Schweizer et al., 1994). Transfection of the mutant’s COS-1 cells led to an increase in tPA-catalyzed plasminogen activation, which confirmed the role of cell surface CKAP4 in tPA binding (Razzaq et al., 2003). Therefore, CKAP4 binds tPA on the surface of VSMC and plays an important role in regulation of the plasminogen activation system in the vessel wall (Table 1).

### Surfactant Protein A

SP-A plays a crucial role in the regulation of the turnover of surfactant lipid by type II pneumocytes. This process, including surfactant secretion and uptake, is involved in the interaction of SP-A with cell surface receptors. Report has confirmed that CKAP4 is an important receptor of SP-A. CKAP4 affects SP-A calcium-dependent (specific) binding to alveolar type II cells and does not affect calcium-independent (nonspecific) binding (Gupta et al., 2006). Interference of SP-A interaction with CKAP4 by using siRNA techniques to reduce the CKAP4 level in the type II cells impacts surfactant secretion (Bates, 2010). Another report showed CKAP4 regulated SP-A-mediated biological activity, stimulating the uptake of surfactant by pneumocytes (Bates et al., 2008). CKAP4 antibody prevented secretagogue-stimulated uptake of liposomes containing SP-A. SP-A regulated the movement of CKAP4 from ER to the plasma membrane in lung epithelial cells through phosphatidylinositol 3-kinase (PI3-kinase) signaling pathway. Blocking the kinase pathway obstructed the intracellular transport of CKAP4 to the cell surface and the resultant alteration of SP-A-mediated lipid turnover (Kazi et al., 2010). These data demonstrate that CKAP4 as SP-A functional receptor is essential for SP-A-mediated interactions with type II lung cells and the regulation of surfactant secretion and uptake (Table 1).

### Alginate Exopolysaccharides

CKAP4 was also found to be a receptor for alginate exopolysaccharides on the surface of airway epithelial cells (AEC) (Barbier et al., 2012). Alginate exopolysaccharides are especially secreted by *Pseudomonas aeruginosa* (PA) during lung infection. CKAP4 as an AEC receptor interacts with alginate-producing strains that mediate bacterial uptake into the cells and increase the binding of purified alginate exopolysaccharide to AEC (Table 1). Previous studies have identified that SP-A is an opsonin that increases PA uptake by alveolar macrophages (Mariencheck et al., 1999). On the contrary, alginates reduce the secretion of SP-A by AEC. CKAP4, as a common and competitive receptor for alginate exopolysaccharides and SP-A, might be a promising drug target for PA infected patients (Barbier et al., 2012; Sandoz and van der Goot, 2015).

### Antiproliferative Factor

CKAP4 was also described as a high-affinity receptor for APF on bladder cells (Conrads et al., 2006). APF is a small sialoglycopeptide secreted by bladder epithelial cells of patients with interstitial cystitis disorder and is a potent inhibitor of both normal bladder epithelial and bladder carcinoma cell proliferation. APF mediates its antiproliferative activity via binding to its receptor CKAP4. A report showed that reducing CKAP4 with siRNA techniques or anti-CKAP4 antibodies reversed the reduction of cell proliferation when cells were exposed to APF (Conrads et al., 2006) (Table 1).

### Dickkopf

The Dickkopf (DKK) family encodes secreted proteins and consists of four main members (DKK1,2,3,4). DKK family proteins contain two conserved cysteine rich domains (CRD-1 and CRD-2). More recently, Kimura and colleagues found that CKAP4 bound to CRD-1 of all DKK family proteins on the apical cell surface membrane of Madin-Darby canine kidney (MDCK) epithelial cells (Kimura et al., 2016). When the DKK proteins bind to CKAP4, the internalization of CKAP4 from the cell membrane is induced (Kajiwara et al., 2018). CKAP4 knockdown or CRD-1 deletion mutants of DKKs inhibited MDCK cellular proliferation (14). These results suggest that CKAP4 as a common receptor of DKK family proteins mediates DKKs-induced cellular proliferation (Table 1).

DKK3 is structurally different from the rest of the DKK family. Early researches showed that DKK3 had many functions, such as regulating cancer cell migration and invasion (Romero et al., 2016) and influencing atherosclerotic plaques (Cheng et al., 2017). CKAP4, as DKK3 receptor, may have the function of regulating other pathological processes other than cell proliferation.

## CKAP4 and Cancer

Many studies have shown that CKAP4 has been related to various cancers including bladder cancer, cervical carcinoma, lung cancer, cholangiocarcinoma, hepatocellular carcinoma, esophageal squamous cell carcinoma, pancreatic cancer, glioma, clear cell renal cell carcinoma, and so on.

### Biological Function of CKAP4 in Cancer

#### Bladder Carcinoma

**FIGURE 2:**
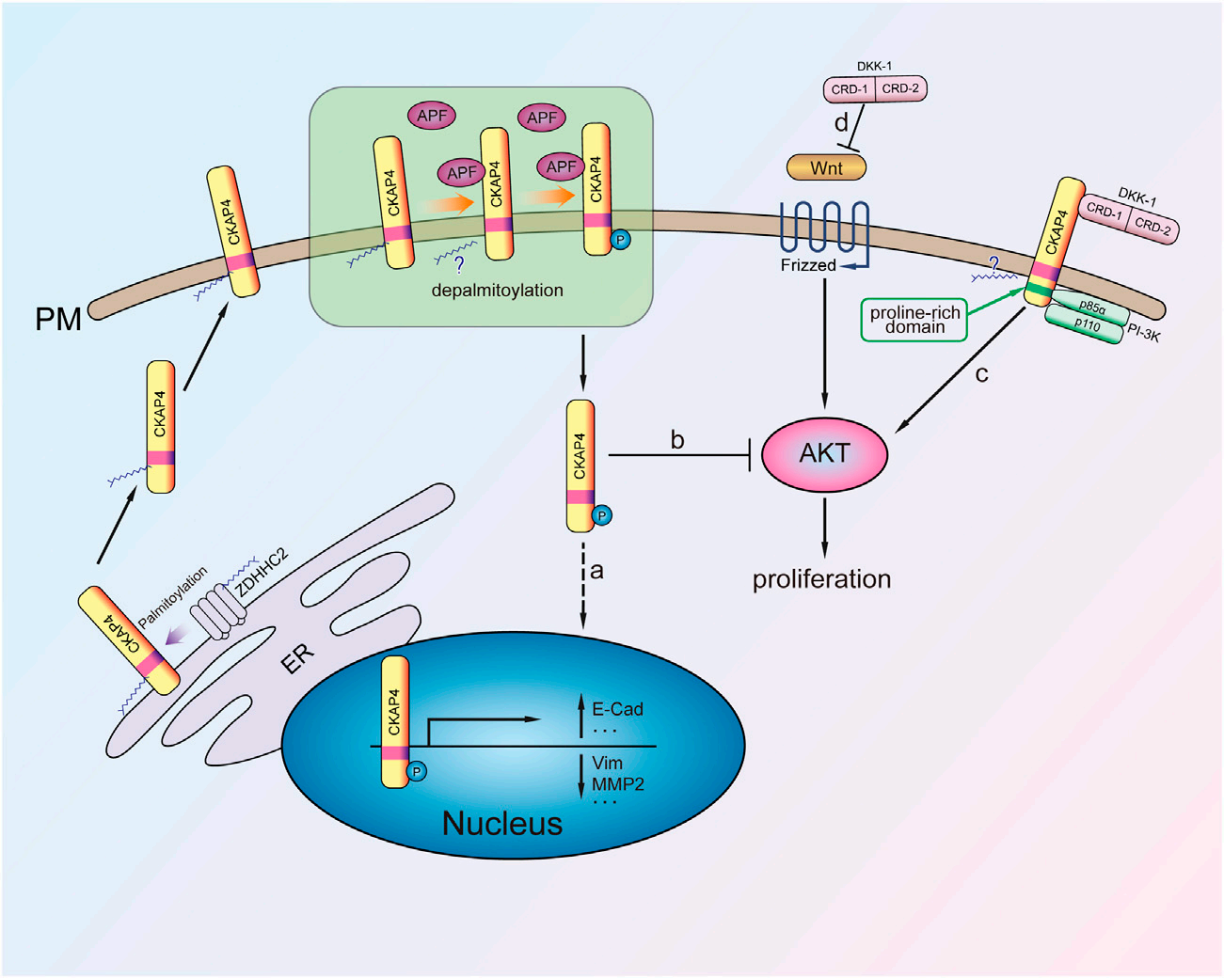
The mechanism of CKAP4 regulating cell proliferation. Palmitoylated CKAP4 translocates from ER to PM. At the PM, CKAP4 binding its ligands APF and DKK-1, CKAP4 might undergo depalmitoylation. a: APF binding to CKAP4 induces phosphorylation of CKAP4, and the phosphorylated CKAP4 translocates to the nucleus, then changes related genes transcription, and plays an antiproliferation effect. b: The phosphorylated CKAP4 decreases AKT phosphorylation and then inhibits proliferation. c: Upon the CRD-1 of DKK-1 binding to CKAP4, CKAP4 binds to the p85α subunit of PI-3K to activate PI3K/AKT signaling and stimulate cell proliferation. d: DKK1 is a potent β-catenin-dependent Wnt signaling antagonist.

CKAP4 as a receptor of APF plays an important role in mediating bladder carcinoma cells proliferation. Following CKAP4 knockdown, the inhibition effect of APF on T24 cell proliferation was eliminated, indicating the important role of the receptor in mediating the antiproliferation activity of APF in bladder cancer cells (Shahjee et al., 2010). Previous study showed that APF could increase p53 expression in T24 bladder carcinoma cells, while p53 knockout by interfering RNA significantly attenuated APF-induced growth stagnation (Kim et al., 2007). The mechanism whereby CKAP4 enhances the inhibitory effect of APF on bladder cancer cell proliferation involves upregulation of tumor suppressor gene p53 expression, along with the activation of AKT/GSK3β/β-catenin phosphorylation and downregulation of MMP2 expression (Shahjee et al., 2010) (Figure 2). In addition, CKAP4 translocated to the nucleus of T24 cells and regulated the CCN2 expression after APF treatment (CCN2 overexpression inhibits invasion and metastasis of the cancer cells both *in vitro* and *in vivo*) (Chen and Lau, 2009). When CKAP4 was reduced with siRNA or anti-CKAP4 antibody, the upregulation of CCN2 during APF treatment was inhibited (Matika et al., 2012). These results confirm that CKAP4 is important for mediating APF’s antiproliferative activity in bladder cancer cells. Another research showed that DKK3, the ligand of CKAP4, was highly expressed in bladder carcinoma cells (Kajiwara et al., 2018). Thus, whether CKAP4 and DKK3 associate with bladder carcinoma prognosis need further studies. In addition, the relationship between the expression of CKAP4 and its ligand DKK3 in bladder cancer tissues and the prognosis is unclear.

#### Cervical Carcinoma

CKAP4 palmitoylation is critical for regulating its subcellular distribution from ER to plasma membrane. Further research demonstrated that CKAP4 phosphorylation of N-terminal serine residues (S3, S17, and S19) is necessary for translocation from the plasma membrane to the nucleus in Hela cell (Zacharias et al., 2012) (Figure 2). It was noted that palmitoylated CKAP4 was not observed in nucleus. As for how palmitoylated CKAP4 becomes depalmitoylated it is unclear. Once in the nucleus, CKAP4 was found to bind DNA through its ectodomain and subsequent changes in the transcription of at least 14 genes (including E-cadherin, vimentin, cyclin D1, p53, and ZO-1) that are involved in regulating proliferation and tumorigenesis (Zhang et al., 2008; Planey et al., 2009) (Figure 2). CKAP4 of palmitoylation site mutant (C100S) or phosphorylation site mutant does not cause relocalization and has APF-like activity inhibiting cell proliferation (Zacharias et al., 2012). It suggests that the mutation of CKAP4 could be used to treat excessive proliferation diseases such as tumor. Another research showed that knockdown of CKAP4 in Hela cells did not affect cellular proliferation for these cells, only expression of CKAP4 but little DKK1 (Kimura et al., 2016). Thus, they considered that expression of CKAP4 and its ligand DKK1 was necessary for cellular proliferation.

#### Hepatocellular Carcinoma

Knockdown of CKAP4 markedly increased cell growth of HCC cells (Li et al., 2014a). Moreover, high expression of CKAP4 inhibited the growth of xenograft tumor and the metastatic potential of HCC in nude mice. These data suggest that CKAP4 is an inhibitor in HCC growth. The mechanism of CKAP4 inhibiting HCC growth and metastasis is that CKAP4 binds extracellular growth factor receptor (EGFR) and associates with EGFR at basal status. Once EGF is present, this complex rapidly dissociates, leading to release of active EGFR into cytoplasm (Li et al., 2014a).

#### Lung Cancer

CAKP4 was considered as an oncogene protein by *in vitro* and *in vivo* experiments about lung cancer. DKK1 has been verified to be a head inducer in *Xenopus* embryos and shown its potent β-catenin-dependent Wnt signaling antagonist (Glinka et al., 1998; Niehrs, 2006) (Figure 2). Downregulation of CKAP4 or DKK1 in A549 lung cancer cells suppressed AKT activity through PI3K. It is noted that the mechanism of CKAP4 binding to PI3K is not the same as other growth factor receptors because CKAP4 has no tyrosine residues. Instead, upon DKK-1 binding, the proline-rich motif of CKAP4 interacts with the SH3 domain of the p85α subunit and then recruits PI3K to its cytoplasmic domain (Figure 2). Through this mechanism, DKK-1 activates AKT and promotes cell proliferation when CKAP4 presents on the cell surface membrane (Bhavanasi et al., 2016). Furthermore, *in vivo* experiments also showed that the low CKAP4 expression cells formed much smaller xenograft tumors than the control group (Kimura et al., 2016).

#### Esophageal Squamous Cell Carcinoma

Research showed that DKK1/DKK3-CKAP4 signaling axis promoted proliferation of ESCC cells through PI3K and AKT pathway. It is worth noting that knockdown of CKAP4 did not influence cell proliferation in cells with insufficient DKK1 and/or DKK3. Therefore, the DKK1 and DKK3 as CKAP4’s ligands are vital for proliferation of ESCC cells (Shinno et al., 2018). The two results were further confirmed *in vivo* by comparing xenograft tumor growth between CKAP4-depeleted TE-8 cells or KYSE960 cells and control cells, respectively (Kajiwara et al., 2018; Shinno et al., 2018).

#### Pancreatic Cancer

In S2-CP8 pancreatic cancer cells, endogenous CKAP4 formed a complex with p85α. When CKAP4 or DKK1 was depleted in S2-CP8 cells, AKT activity and cellular proliferation were suppressed. CKAP4 or DKK1 knockdown also reduced invasion and migration activity of SC-CP8 cells. Therefore, CKAP4 mediated pancreatic cancer cell proliferation by PI3K/AKT signal pathway (Kimura et al., 2016). Another study showed CKAP4 not only regulated the migration of pancreatic cancer cell by relying on DKK1, but also mediated integrin trafficking independently of DKK1 (Osugi et al., 2019). Endogenous β1 integrin was CKAP4-binding protein and they interacted with each other in the cell surface and cytoplasm (Osugi et al., 2019). CKAP4 promoted cell migration and reduced cell adhesion sites through decreasing α5 integrin level in the total cell lysates. It was noted that CKAP4 was not directly involved in α5β1 integrin endocytosis but inhibited its recycling (Osugi et al., 2019). Taken together, CKAP4 regulated pancreatic cancer cell adhesion and migration through interaction with and recycling of α5β1 integrin.

#### Glioma

The expression of miR-671-3p, which promotes proliferation and migration in glioma cells, was high in glioma tissues and its target gene CKAP4 was of low expression in glioma tissues compared with the corresponding noncancerous tissues (Lu et al., 2018). In addition, the expression of CKAP4 was also detected to be downregulated in astrocytoma and glioblastoma samples. Reversal experiments confirmed that CKAP4 overexpression inhibited the proliferation and migration effects of miR-671-3pin glioma cells (Lu et al., 2018). Therefore, CKAP4, as a tumor suppressor, negatively regulated the progression of glioma.

#### Other Cancers

CKAP4 overexpression promoted cell proliferation, invasion, and migration of clear cell renal cell carcinoma (ccRCC) cells. Mechanistically, CKAP4 was revealed to play a role in G2/M phase of cell cycle through CCNB signaling in ccRCC (Sun et al., 2017). CKAP4 was also observed to demonstrate overexpression in tumors of mouse model of CLL and chronic lymphocytic leukemia (CLL) patients (Johnston et al., 2017; Johnston et al., 2018). However, the specific role of CKAP4 in CLL needs further study. Research in malignant mesothelioma (MM) cells has shown that expression of both DKK1 and CKAP4 is necessary for MM cell proliferation (Sato et al., 2018). Another research confirmed that CKAP4 expression was significantly elevated in tissue samples from patients with meningioma (Dunn et al., 2019). It should be noted that EGFR, as an affinity protein of CKAP4, was also significantly overexpressed in meningioma (Dunn et al., 2019). Butthe biological function of CKAP4 in meningioma is still unclear and needs further study.

### CKAP4 and Cancers Prognosis

#### Hepatocellular Carcinoma

Our group investigated the relationship between CKAP4/DHHC2 expression and prognosis in 416 patients with hepatocellular carcinoma (HCC) (Li et al., 2014b) (Table 2). The results showed that the expression of CKAP4 in HCC tumor tissues was significantly higher than that in para-carcinoma normal tissues. Meanwhile, DHHC2 examination showed that its expression was low in 87.6% of these cases. Prognosis was favorable in patients with high CKAP4 or high DHHC2 expression compared with those with low CKAP4 or low DHHC2 expression. The prognosis was the best when carcinoma cells presented high amount of CKAP4 and high expression level of DHHC2. When considering protein expression of both CKAP4 and DHHC2 in HCC, prognosis accuracy was improved compared to considering only either of the two proteins (Li et al., 2014b).

**TABLE 2:** Relationship between CKAP4 protein expression and various cancers prognosis.

Cancer types	Clinical tissue samples	CKAP4 positive rate (%)	Ligand positive rate (%)	Clinicopathological parameters^a^	Prognosis	References
ICC	173	84.7	—	Lymph node metastasis	Positive	19
HCC	416	61 (low expression DHHC2: 87.6)	—	Tumor size, lymph node metastasis, UICC, and TNM stage features	Positive (high expression of CKAP4 and DHHC2 has the best prognosis)	20
Lung cancer	67 adenocarcinoma	74.6	DKK1 79.1	a	Negative	15
	61 squamous cell carcinoma	74.6	DKK1 73.8			
ESCC	119	55.5	DKK1 52.1 (both CKAP4 and DKK1 positive: 38.7)	a	Negative (high expression of CKAP4 and DKK1 has the worst prognosis)	17
	72	65.2	DKK3 51.4 (both CKAP4 and DKK3 positive: 37.5)	a	Negative (high expression of CKAP4 and DKK3 has the worst prognosis)	14
PDAC	59	66.1	DKK1 76.3 (both DKK1 and CKAP4 positive: 35.6)	a (DKK1 was associated with perineural invasion of the tumor)	Negative	15
ccRCC	124	—	—	T, N, M stages and Fuhrman grades	Negative	18

ccRCC, clear cell renal cell carcinoma; ESCC, esophageal squamous cell carcinoma; HCC, hepatocellular carcinoma; ICC, intrahepatic cholangiocellular carcinoma; PDAC, pancreatic ductal adenocarcinoma.

^a^ The correlation of CKAP4 expression and clinicopathological parameters, including tumor size, tumor T, N, M grade, tumor invasion, lymph node metastasis, or venous invasion.

a, CKAP4 expression was not correlated with clinicopathological parameters.

—: no relevant data.

#### Cholangiocarcinoma

CKAP4 has been related to intrahepatic cholangiocellular carcinoma (ICC) prognosis (Table 2). We analyzed 173 ICC patient tissues; the results revealed that CKAP4 overexpression was observed in the great mass of ICC (strong expression 20.3%; moderate expression 37.2%; weak expression 26.7%) and associated with distant metastasis and lymph node metastasis (Li et al., 2013). Prognostic values of CKAP4 expression showed that the high-CKAP4 patients had a much longer overall survival and lower recurrence rate than the low-CKAP4 patients, and CKAP4 is an independent predictor for overall survival in ICC patients (Li et al., 2013). Then, CKAP4 might serve as a promising prognostic biomarker in patients with ICC. Further research is needed to better understand the mechanism of CKAP4’s involvement in ICC development.

#### Lung Cancer

Nagashio and coworkers observed that CKAP4 was expressed in fibroblasts of the tumor stroma and the cytoplasm of tumor cells but not in normal lung specimens in tissue chips containing 70 consecutive pairs of lung cancers (Yanagita et al., 2018). Kimura group analyzed the values of CKAP4 and its ligand DKK1 on prognosis in 67 cases of lung adenocarcinoma and 61 cases of squamous cell carcinoma specimens (Kimura et al., 2016). The positive rates of CKAP4 and DKK1in lung adenocarcinoma were 74.6 and 79.1%, respectively, and in squamous cell carcinoma were 74.6 and 73.8%, respectively (Kimura et al., 2016) (Table 2). Patients positive for both CKAP4 and DKK1 showed a more shorter relapse-free survival than patients positive for either CKAP4 or DKK1 or negative for both (Kimura et al., 2016) (Table 2). In this study, overall survival had not been examined for insufficient follow-up period. In addition, CKAP4 was minimally detected in 11 cases of atypical adenomatous hyperplasia. It is worth noting that there were approximately 50% DKK1 positive in such putative precursor lesion of adenocarcinoma cases (Kimura et al., 2016). It suggested that CKAP4 expression indicates transition from precancerous to cancerous state. It also can be speculated that the expression of CKAP4 is more sensitive than its ligand DKK1 in distinguishing tumor tissues from nontumor tissues.

#### Esophageal Squamous Cell Carcinoma

Shinno et al. and AndKajiwara et al. analyzed 119 cases and 72 cases of esophageal squamous cell carcinoma (ESCC) and showed that expression of CKAP4, along with DKK1 and/or DKK3, in ESCC was associated with poor prognosis (Kajiwara et al., 2018; Shinno et al., 2018) (Table 2). In 119 tumor lesions of ESCC, there were close to 40% of cases positive for both DKK1 and CKAP4, about 30% of cases positive for either CKAP4 or DKK1, and approximate 30% negative for both (Shinno et al., 2018). In Kajiwara’s study, CKAP4 and DKK3 were positively detected in 65.2 and 51.4% of cases, respectively, whereas positive expression of both was minimally detected in nontumor regions (Kajiwara et al., 2018). The two studies show that the expression of DKK1 and DKK3 was all significantly higher in the tumor lesions than in the nontumor regions. However, the two ligands are separately expressed in different tumor lesions in the same cases and in the different ESCC cases and cultured ESCC cells. Therefore, the DKK1-CKAP4 and DKK3-CKAP4 axes might be activated in different ESCC cell populations (Kajiwara et al., 2018). Furthermore, they analyzed the positive expression rates of DKK1 and DKK3 in ESCC and found there were 54.2% of ESCC cases expressing CKAP4 and its ligands (DKK1 40.3%, DKK3 37.5%, and DKK1 and DKK3 23.6%) (Kajiwara et al., 2018). Patients positive for both CKAP4 and ligands (DKK1 and/or DKK3) showed unfavorable prognosis and shortened relapse-free survival than do patients positive for either CKAP4 or DKK1/DKK3 or negative for CKAP4 and DKK1/DKK3 (Kajiwara et al., 2018; Shinno et al., 2018) (Table 2).

#### Pancreatic Cancer

Kimura et al. investigated the expression of CKAP4 and DKK1 in 59 cases of pancreatic ductal adenocarcinoma (PDAC) (Kimura et al., 2016). CKAP4 and DKK1overexpression were observed in approximately 70% of cases. There was no correlation between the expression of the DKK1 or CKAP4 and clinicopathological parameters, except perineural invasion associated with positive DKK1 (Table 2). In addition, the expression of CKAP4 and DKK1 was negatively correlated with overall survival and relapse-free survival in PDAC (Kimura et al., 2016; Kikuchi et al., 2017).

#### Clear Cell Renal Cell Carcinoma

Sun and coworkers reported that overexpression of CKAP4 occurred in only 5% of ccRCC patients. However, patients with high CKAP4 protein levels had unfavorable overall survival and relapse-free survival, and overexpression of CKAP4 was significantly associated with TNM stages and Fuhrman grades (Sun et al., 2017) (Table 2).

The above studies show that CKAP4 is closely related to the development of tumors, but the function of CAKP4 in various cancers is controversial. Based on the current research, several possible reasons are proposed as follows. Firstly, the function of CKAP4 in tumors may be related to its binding ligand. From the present studies, we can speculate that CKAP4 can combine with its ligand to affect cancer growth. In the cells with high CKAP4 but with little ligands, DKKs, CKAP4 did not affect cellular proliferation (Kimura et al., 2016; Shinno et al., 2018). On the other hand, patients positive for both CKAP4 and DKKs show unfavorable prognosis and shorter relapse-free survival in pancreatic, lung, and esophageal tumors (Kimura et al., 2016; Kajiwara et al., 2018; Shinno et al., 2018). However, high expression of CKAP4 showed a favorable overall survival and longer disease-free survival in HCC and ICC (Li et al., 2013; Li et al., 2014b). Since CKAP4’s ligand has not been examined in HCC and ICC, further studies are needed to determine the ligands of CKAP4.

Secondly, palmitoylation may affect the role of CKAP4 in tumors. Palmitoylated CKAP4 translocates to the nucleus and then binds to CCN2 gene, which inhibits cell proliferation, adhesion, migration, differentiation, and survival (Zhang et al., 2008; Zacharias et al., 2012). Palmitoylation-deficient CKAP4 mutant was unable to complete intracellular translocation, thus losing the corresponding function. In HCC, it revealed that CKAP4 expression was high while its palmitoyl-transferase DHHC2 expression was low in most tumor tissues, and high expression of CKAP4 and DHHC2 was positive with prognosis (Li et al., 2014b). Whether the palmitoylation site of CKAP4 is mutated in tumor tissues and the expression change of DHHC2 in other tumors require further investigation.

Another possible reason is that different types of tumors have specific microenvironments that trigger activation of specific signaling pathways. DKK1/DKK3-CKAP4 signaling axis was shown to promote progression of ESCC, lung cancer, and pancreatic cancer through PI3K and AKT pathway (Kimura et al., 2016; Kikuchi et al., 2017; Kajiwara et al., 2018). On the other hand, CKAP4 mediated integrin trafficking independently of DKK1 and affected pancreatic cancer cell migration (Osugi et al., 2019). Another report considered CKAP4 as a promotion protein on cancer progression through CCNB signaling in ccRCC (Sun et al., 2017). Meanwhile, different mechanisms have been proposed that CKAP4 is an anticancer protein. For example, CKAP4 inhibited EGFR activation in liver cancer and suppressed miR-671-3p induced cell proliferation and migration in glioma (Li et al., 2014a; Lu et al., 2018).

In general, CKAP4 is widely expressed in various types of tumors. However, the status of CKAP4 is affected by many aspects, such as palmitoylation and phosphorylation; the role of CKAP4 in tumors is dynamically regulated under specific circumstance. Therefore, in further study, the function of CKAP4 in special type of tumor should be analyzed individually.

### CKAP4 as a Serological Marker of Cancer

Previous studies reported that CKAP4 as an ER enriched protein could be secreted by exosomes of ovarian and colon cancer cells and detected as an exosomal protein in urine (Gonzales et al., 2009; Demory Beckler et al., 2013; Liang et al., 2013). CKAP4 might be secreted into the serum in the same manner as urinary exosomal protein. At present, there have been studies examining the serum levels of CKAP4 in various cancers (Table 3), including hepatocellular carcinoma (Li et al., 2016; Wang et al., 2019), lung cancer (Yanagita et al., 2018), pancreatic cancer (Kimura et al., 2019), and ESCC (Chen et al., 2018).

**TABLE 3:** CKAP4 as a serological marker in various cancers.

Cancer types	Clinical samples	AUC	Specificity (%)	Sensitivity (%)	References
HCC	Normal: 40	0.771	67.6	70	22
	HCC: 90				
	HCC: 100	0.821 (0.936 combined AFP)	67 (96.3 combined AFP)	79 (80 combined AFP)	26
	Healthy control: 100				
Lung cancer	Lung cancer patients: 271	0.89	86.0	81.8	24
	Healthy control: 100				
PDAC	PDAC: 47	Exosome ELISA: 0.785 sandwich ELISA: 0.804	—	—	25
	Healthy control: 18				
ESCC	ESCC: 207	0.675	99.5	41.5	59
	Healthy control: 207				

AUC, area under curve; ICC, intrahepatic cholangiocellular carcinoma; HCC, hepatocellular carcinoma; PDAC, pancreatic ductal adenocarcinoma; ESCC, esophageal squamous cell carcinoma; AFP, alpha-fetoprotein; —, no relevant data.

#### Hepatocellular Carcinoma

Our group found that CKAP4 could be detected in serum of HCC patients by coimmunoprecipitation assays (Li et al., 2016) (Table 3). Using serum samples of 40 healthy volunteers and 90 patients with HCC, serological diagnostic efficacy of CKAP4 was examined. The results showed that the sensitivity of the serum CKAP4 is 70% and the specificity is 67.5%, and detection of serum CKAP4 increased the diagnostic efficacy especially in HCC patients with low or negative alpha-fetoprotein (AFP) (Li et al., 2016).

Recently, another group confirmed our result by analyzing the value of CKAP4 as a serological diagnostic marker for HCC. Serum levels of CKAP4 were significantly higher in patients with HCC than in ones with chronic hepatitis B (CHB) infection and cirrhosis and healthy controls (Wang et al., 2019). The sensitivity and specificity for HCC diagnosis of CKAP4 alone had no advantage compared with AFP, but combined CKAP4 and AFP showed a better diagnostic accuracy (sensitivity = 0.8, specificity = 0.963), even in early HCC (sensitivity = 0.762, specificity = 0.963), which was similar to our results (Wang et al., 2019) (Table 3).

#### Lung Cancer

Nagashio et al. generated monoclonal antibodies using A549 cells as an immunogen and developed markers for early and/or differential diagnostics of pulmonary adenocarcinomas (Nagashio et al., 2010). One of the antibodies, only reacting with tumor cells in lung tumor tissues, was recognized to interact with CKAP4 (Yanagita et al., 2018). Further, they analyzed CKAP4 diagnostic value in 271 lung cancer patients and 100 healthy controls (Yanagita et al., 2018). The results showed that the serum CKAP4 levels were obviously higher in lung cancer patients than in healthy persons. The sensitivity and specificity for lung cancer diagnosis were 81.1% and 86.0%, respectively (Table 3), which were higher than other serodiagnostic markers (including carcinoma embryonic antigen, squamous cell carcinoma antigen, and cytokeratin 19 fragment). Furthermore, serum CKAP4 sensitivity was also high even in early stage disease. On the other side, the serum levels of CKAP4 were also of relationship with distant metastasis of lung adenocarcinoma patients (Yanagita et al., 2018).

#### Pancreatic Cancer

Kimura and coworkers found that CKAP4 protein can be secreted with small extracellular vesicles (SEVs) and reflect cell surface expression of CKAP4 in cancer cells (Kimura et al., 2019). Then they confirmed that CKAP4 was released with exosomes from pancreatic ductal adenocarcinoma (PDAC) cells. Serum CKAP4 measured by two types of ELISAs: an exosome ELISA and a sandwich ELISA, was significantly higher in immunohistochemically CKAP4-positive cases than those of negative cases. ROC analysis showed that the area under the curve (AUC) was 0.785 and 0.804 in exosome ELISA and sandwich ELISA, respectively (Table 3). There is no clear difference in diagnosis capacities between two ELISAs. In 47 PDAC patients, including 27 resectable and 20 unresectable cases, the serum levels of CKAP4 were obviously higher in unresectable cases than in resectable ones (Kimura et al., 2019), suggesting serological CKAP4 correlated with tumor stages. It was noted that CKAP4 was higher in preoperative sera, whereas it was greatly reduced in postoperative sera (Kimura et al., 2019). The results showed that serological level of CKAP4 is directly related to the tumor lesions and CKAP4 secreted with exosomes into serum may represent a biomarker for pancreatic cancer.

#### Esophageal Squamous Cell Carcinoma (ESCC)

Chen’s group revealed that the serum levels of CKAP4 were higher in ESCC patients than healthy controls. The sensitivity and specificity for diagnosis ESCC were 0.415 and 0.995, respectively (Chen et al., 2018) (Table 3). Further study showed that the serum levels of CKAP4 were negatively associated with relapse-free survival (Chen et al., 2018), which suggested CKAP4 might be a useful serodiagnostic marker not only for diagnosis ESCC but also for prediction of clinical prognosis of ESCC.

Taken together, these studies have showed that CKAP4 is expected to be a serological marker for HCC (Wang et al., 2019), lung cancer (Yanagita et al., 2018), PDAC (Kimura et al., 2019), and ESCC (Chen et al., 2018). In view of previous findings, the serum content of CKAP4 in ICC (Li et al., 2013), renal cancer (Sun et al., 2017), glioma (Lu et al., 2018), bladder cancer (Shahjee et al., 2010), and other cancers should be evaluated and its diagnostic value should be assessed in further studies. In addition, the sample size of present publications was relatively small. Therefore, large samples and multicenter studies are needed to confirm whether CKAP4 can be used as a serologic marker to diagnose tumor and predict prognosis. The mechanisms about how CKAP4 as a transmembrane protein is secreted into the serum is under investigation. A recent study has revealed that CKAP4 was released with exosomes from pancreatic ductal adenocarcinoma cells (Kimura et al., 2019). Previous research showed that exosomes can be detected in urine, saliva, plasma, and other body fluids (Liang et al., 2013). So, detecting CKAP4-containing exosomes in other body fluids, such as ascites and hydrothorax, holds a significant potential as an adjuvant to the diagnosis of cancer in a noninvasive manner. In addition, whether there are other mechanisms involved in the secretion of CKAP4 into serum still needs further study.

### CKAP4 as a Molecular Target for Cancer Therapy

Sufficient studies have confirmed that CKAP4 correlated with various cancers’ progress and prognosis. The formation of the xenograft tumors derived from CKAP4-depleted cells, including lung cancer cells (Kimura et al., 2016), pancreatic cancer cell (Kimura et al., 2016), and ESCC cells (Shinno et al., 2018), was much less than that of control tumors. Furthermore, an anti-CKAP4 polyclonal antibody (pAb) can significantly reduce xenograft tumor volume and weight caused by these cancer cell lines, suggesting that CAKP4 represents a novel molecular target for cancer therapy (Kimura et al., 2016; Kimura et al., 2019). Kimura et al. generated mouse anti-CKAP4 mAb based on the mouse medial iliac lymph node method and spleen method. The results showed that CKAP4 mAbs inhibited metastasis of PDAC cells and extended survival of mice via inhibition of DKK1-CKAP4 signaling (Kimura et al., 2019). They also generated CKAP4 knockout (KO) mice and observed that the CKAP4-KO mice were fertile and survived for at least 18 months. In addition, various organs in CKAP4 KO mice did not show any differences from wild type (WT) mice, which means loss of CKAP4 function by anti-CKAP4 mAb will not affect critical cellular functions (Kimura et al., 2019). Thus, CKAP4 antibody has a great prospect in the treatment of some types of tumors.

### CKAP4 and Noncancers

Although plentiful researches focused on the relationship between CKAP4 and cancer, there were also studies that showed that CKAP4 played an important role in noncancers. One study revealed that CKAP4 was a gentamicin-binding protein by mass spectrometry and involved in drug-induced cytotoxicity (Karasawa et al., 2010). Proximal tubule cells and cochlear cell lines expressed greater levels of monomeric CKAP4 and dithiothreitol- (DTT-) resistant dimers. DTT-resistant CKAP4 dimers, which were induced by gentamicin and required CKAP4 palmitoylation, were vital for CKAP4-dependent gentamicin cytotoxicity (Karasawa et al., 2010). Further research showed that the cytosolic domain of CKAP4 bound to 14-3-3 proteins (Karasawa et al., 2010). Gentamicin may cause cell death by interfering with cell proliferation through CKAP4 and 14-3-3 proteins.

CKAP4 was also identified as a novel marker for activated fibroblasts by using single-cell sequencing, and that positively correlates with known myofibroblast markers in diseased hearts of murine and human (Gladka et al., 2018). Mechanistically, TGF-β was revealed to induce CKAP4 expression, and inhibition of CKAP4 increased the expression of markers for activated fibroblasts under TGF-β treatment (Gladka et al., 2018). This seemed to imply that CKAP4 in activated fibroblast inhibited the expression of genes of activated fibroblast.

CKAP4, which binds to its ligand APF, could promote excessive proliferation of bladder epithelial cells and induce interstitial cystitis/painful bladder syndrome (IC/PBS) (Conrads et al., 2006). The potential mechanism may be associated with CKAP4 palmitoylation and phosphorylation (Zacharias et al., 2012). Another research showed that it is possible to establish a noninvasion diagnostic method for IC/PBS because the special domains of CKAP4 (AA127-360, AA361-524) could enhance the binding activity to APF, increasing detection efficiency to APF concentrations in urine (Chavda et al., 2017). It is possible that inhibiting the binding of CKAP4 and APF may be an effective strategy for the treatment of IC/PBS-related bladder pathology.

Laminar shear stress (LSS) determines endothelial cell (EC) homeostasis and confers protection against vascular diseases such as atherosclerosis (Davies, 2009). SENCR is an LSS-induced long-noncoding RNA (lncRNA) that regulates VSMC and EC phenotypes. Qing Lyu et al. found that CKAP4 was a SENCR-binding protein using biotinylated RNA pull-down and mass spectrometry and played an important role in promotion of EC adherens junction integrity (Lyu et al., 2019). SENCR affected CKAP4 anchor at the ER membrane. Therefore, the precise function of the SENCR–CKAP4 complex needs further study to confirm.

## Conclusion

CKAP4 is a protein predominantly localized to the ER. The cytoplasmic domain of CKAP4 could anchor ER and bind microtubule. It plays an important role in regulating ER street. CKAP4 is also a receptor of tPA/SP-A/AFP/alginate exopolysaccharide/DKKs at the different cell surface. In various tumors, CKAP4 could be used as an anticancer protein to inhibit tumor progression, such as HCC, ICC, and glioma. Otherwise, it could be as a protumor molecule to promote progression of lung, pancreatic, esophageal, and kidney cancers. Moreover, CKAP4 is secreted into the serum with exosomes in tumors and is expected to be a novel serological marker for diagnosis of HCC, lung cancer, PDAC, and ESCC. Considering the function of CKAP4 in various tumors, recent studies suggest that CKAP4 may be a therapeutic target for tumors. The multifunction of CKAP4 is not only in tumors, but also in some nontumors, such as fibrosis. In line with plenty of researches, many roles of CKAP4 have been discovered, but many controversial issues also emerged, which made us more confused about this mysterious molecule. Therefore, further studies specifically uncovering more molecular functions of CKAP4 will help us to understand these discrepancies.

## Abbreviations

ACE, airway epithelial cells; APF, antiproliferative factor; ccRCC, clear cell renal cell carcinoma; CHB, chronic hepatitis B; C/PBS, interstitial cystitis/painful bladder syndrome; CKAP4, cytoskeleton-associated protein 4; CLIMP-63, cytoskeleton-linking membrane protein; CRD, cysteine rich domains; DKKs, Dickkopfs; DTT, dithiothreitol; EGF, extracellular growth factor; EGF-R, extracellular growth factor receptor; ELISA, enzyme-linked immunosorbent assay; ER, endoplasmic reticulum; ESCC, esophageal squamous cell carcinoma; HCC, hepatocellular carcinoma; ICC, intrahepatic cholangiocarcinoma; IEC, endothelial cell; LSS, laminar shear stress; PA, *Pseudomonas aeruginosa*; PDAC, pancreatic ductal adenocarcinoma; PI3K, phosphatidylinositol-3 kinase; SEVs, small extracellular vesicles; SP-A, surfactant protein A; tPA, tissue plasminogen activator; VSMC, vascular smooth muscle cells.
